# The S-Connect study: results from a randomized, controlled trial of Souvenaid in mild-to-moderate Alzheimer’s disease

**DOI:** 10.1186/alzrt224

**Published:** 2013-11-26

**Authors:** Raj C Shah, Patrick J Kamphuis, Sue Leurgans, Sophie H Swinkels, Carl H Sadowsky, Anke Bongers, Stephen A Rappaport, Joseph F Quinn, Rico L Wieggers, Philip Scheltens, David A Bennett

**Affiliations:** 1 Rush Alzheimer’s Disease Center, Rush University Medical Center, 600 South Paulina, Suite 1022, Chicago, IL 60612, USA; 2 Nutricia Advanced Medical Nutrition, Nutricia Research, Uppsalalaan 12, PO Box 80141, 3584TC, Utrecht, the Netherlands; 3 Nutricia Research, Uppsalalaan 12, PO Box 80141, 3584TC, Utrecht, the Netherlands; 4 Department of Neurology, Nova Southeastern University, 33407 North Congress Avenue, West Palm Beach FL, USA; 5 Agewell Health®, 46260 North Meridian Street, Indianapolis IN, USA; 6 Department of Neurology, Oregon Health and Science University and the Portland VA Medical Center, 3181 Sw Sam Jackson Park Road, Portland OR, USA; 7 Alzheimer Center, VU University Medical Center, PO Box 7057, 1007MB, Amsterdam, the Netherlands

## Abstract

**Introduction:**

Souvenaid® containing Fortasyn® Connect is a medical food designed to support synapse synthesis in persons with Alzheimer’s disease (AD). Fortasyn Connect includes precursors (uridine monophosphate; choline; phospholipids; eicosapentaenoic acid; docosahexaenoic acid) and cofactors (vitamins E, C, B12, and B6; folic acid; selenium) for the formation of neuronal membranes. Whether Souvenaid slows cognitive decline in treated persons with mild-to-moderate AD has not been addressed.

**Methods:**

In a 24-week, double-masked clinical trial at 48 clinical centers, 527 participants taking AD medications [52% women, mean age 76.7 years (Standard Deviation, SD = 8.2), and mean Mini-Mental State Examination score 19.5 (SD = 3.1, range 14–24)] were randomized 1:1 to daily, 125-mL (125 kcal), oral intake of the active product (Souvenaid) or an iso-caloric control. The primary outcome of cognition was assessed by the 11-item Alzheimer’s Disease Assessment Scale-Cognitive Subscale (ADAS-cog). Compliance was calculated from daily diary recordings of product intake. Statistical analyses were performed using mixed models for repeated measures.

**Results:**

Cognitive performance as assessed by ADAS-cog showed decline over time in both control and active study groups, with no significant difference between study groups (difference =0.37 points, Standard Error, SE = 0.57, p = 0.513). No group differences in adverse event rates were found and no clinically relevant differences in blood safety parameters were noted. Overall compliance was high (94.1% [active] and 94.5% [control]), which was confirmed by significant changes in blood (nutritional) biomarkers.

**Conclusions:**

Add-on intake of Souvenaid during 24 weeks did not slow cognitive decline in persons treated for mild-to-moderate AD. Souvenaid was well tolerated in combination with standard care AD medications.

**Trial registration:**

Dutch Trial Register number: NTR1683.

## Introduction

By 2050 the number of individuals living with dementia due to Alzheimer’s disease (AD) worldwide is estimated to increase from 36 million to 115 million people [1], with two-thirds of persons affected living in developing countries. Given the worldwide public health impact of AD, increased efforts are needed to develop novel and effective AD interventions that are easy to deploy and are not resource intensive. AD is a neurodegenerative condition associated with cognitive and functional ability loss. While the pathogenesis of AD involves the extraneuronal deposition of the amyloid-beta peptide and phosphorylation of intraneuronal tau proteins [2], loss of synapses is thought to play an important downstream role in the process of cognitive loss [3,4]. The investigational nutrition product, Souvenaid (Nutricia N.V., Zoetermeer, the Netherlands), is a liquid medical food formulation containing the specific nutrient combination, Fortasyn Connect (Nutricia N.V.). Fortasyn Connect includes nutritional precursors and cofactors for the synthesis of neuronal membranes and is designed to support synapse formation and function in patients with AD [5]. Phosphatide molecules plus synaptic proteins comprise the bulk of synaptic membranes and can be increased by co-administration of rate-limiting precursors via the Kennedy pathway [6,7].

In a multicenter, European, randomized, double-blind, controlled proof-of-concept trial (Souvenir I), 225 drug-naïve patients with mild AD were randomized to once-daily intake of Souvenaid or control [8]. In this trial, delayed verbal recall score of the Wechsler Memory Scale – revised was significantly improved after 12 weeks of intervention with Souvenaid as compared with control product. The 13-item modified Alzheimer’s Disease Assessment Scale – Cognitive Subscale (ADAS-cog) score, the other co-primary outcome, was no different in the Souvenaid group compared with the control group, but secondary analyses pointed to a potential benefit in individuals with worse baseline performance on the ADAS-cog [9]. Based on these results, two double-blind, randomized controlled clinical trials were designed. The Souvenir II study examined the effect of longer treatment duration (24 weeks) with Souvenaid as compared with control product on memory performance in drug-naïve mild AD [10]. Since the ADAS-cog may be more sensitive to change in moderate AD [11] and since Souvenaid had not been tested in moderate AD patients already taking AD medications, the S-Connect study was designed. In this 24-week, double-masked, parallel, randomized, controlled clinical study, the efficacy and tolerability of Souvenaid was investigated in 527 persons with mild-to-moderate AD taking stable doses of US Food and Drug Administration-approved symptomatic AD treatments (that is, cholinesterase inhibitors and/or memantine), using the ADAS-cog as the primary outcome measure. The results of the S-Connect study are presented here.

## Methods

### Standard protocol approvals, registrations, and patient consents

The S-Connect study was approved by the Institutional Review Boards of each of the 48 clinical sites based in the United States. The study was conducted in accordance with the Declaration of Helsinki, the International Conference on Harmonisation guidelines for Good Clinical Practice as appropriate for nutritional products, and local legislation of the country in which the research was conducted. The trial was registered with The Dutch National Trial Register (NTR1683). Written informed consent was obtained from all study participants and study partners prior to conducting study procedures.

### Patients

Community and clinic-based recruitment efforts including mass-media presentations in certain markets that received Institutional Review Board approval were utilized to identify potential participants. Persons expressing interest in the study were invited for a screening evaluation. Screening involved confirmation of eligibility criteria via the collection of demographic information, medical history and concomitant medications, and the administration of the Mini-Mental State Examination (MMSE) [12]. Inclusion criteria were: age 50 years or older; diagnosis of probable AD according to the joint working group of the National Institute of Neurological and Communicative Disorders and Stroke and the Alzheimer’s Disease and Related Disorders Association [13]; a MMSE score between 14 and 24 inclusive; use of US Food and Drug Administration-approved AD medication on a stable dose for at least 4 months prior to baseline; and availability of a responsible study partner. Exclusion criteria were: diagnosis of a neurological/psychiatric disease significantly contributing to cognitive difficulties other than AD; a 15-item Geriatric Depression Scale [14] score >4; recent use of potent anticholinergic agents, antipsychotics, omega-3 fatty acid-containing supplements and/or oily fish consumption more than twice a week, high-energy or high-protein nutritional supplements or medical foods, vitamins B, C and/or E containing supplements at >100% of daily value, or other investigational products; recent change in lipid-lowering medications, antidepressants, or antihypertensives; alcohol or drug abuse in the opinion of the investigator; or institutionalization in a nursing home. Participants who discontinued the study prematurely were not replaced.

### Study group allocation

Participants meeting eligibility criteria at baseline were randomized in a 1:1 fashion to active product (Souvenaid containing Fortasyn Connect) or an iso-caloric control product that lacked Fortasyn Connect but was similar in appearance and taste with the active product (see Additional file 1 for detailed product composition). Both study products were available in two flavors (strawberry or vanilla) as a 125 ml (125 kcal) drink in a tetra package and were to be taken once daily for 24 weeks. Participants chose one of the two flavors based on personal taste preferences. Allocation to active or control product was performed through a central randomization procedure in the Electronic Data Capture system using four different randomization codes (A, B, C, and D). Participants, study partners, and study staff were masked to study group assignment during the trial. Unmasking did not occur until initial statistical modeling of the primary outcome was complete.

### Procedures

Participants underwent a baseline visit that included functional evaluation and global clinician rating. The main efficacy outcome and secondary outcomes were measured at baseline, 12 and 24 weeks, except for the blood parameters that were assessed at baseline and 24 weeks. Additional brief evaluations occurred at weeks 6 and 18. Telephone calls to participants/caregivers by study staff were conducted at 3, 9, 15, and 21 weeks as well as 2 weeks after completion. Adverse events and the use of concomitant medication, including AD medication, were recorded at every in-person and telephone evaluation. All participants who withdrew early had study termination visits equivalent to week 24.

### Primary outcome

The primary outcome measure was the effect on cognition of the active product as measured by the 11-item ADAS-cog [15]. The ADAS-cog assesses memory, language, praxis, attention, and other cognitive abilities. The total ADAS-cog score ranges from 0 (no cognitive deficit) to 70 (severe cognitive deficit), calculated as the numbers of errors a participant made. All examiners were trained centrally before conducting the tests for the primary and secondary outcome assessments. While no formal continuing training program during the study was implemented, additional training was provided as needed if cognitive data collection issues were noted by centrally trained study monitors. No screening or practice sessions for cognitive outcomes were conducted prior to baseline.

### Secondary outcomes

The secondary outcomes assessed the effect on cognition, functional abilities, global clinical impression, safety, and nutritional blood parameters of the active product as compared with control.

To assess cognition in a complementary manner to the ADAS-cog, a cognitive test battery composed of the Digit Span from the Wechsler Memory Scale – Third Edition [16], the Concept Shifting Test [17], the Letter Digit Substitution Test [18], and Category Fluency [19] was administered to measure attention and concentration, executive function, processing speed, and semantic memory, respectively. The total score on the Digit Span tests was calculated as the total number of Digits Forward and Digits Backward sequences correctly repeated (ranging from 0 to 24). The Concept Shifting Test score was calculated from the time needed to complete each of the subtests, resulting in a concept shifting score. The Letter Digit Substitution Test score was the total number of correctly substituted numbers in 60 seconds. The Category Fluency score was the total number of different animals named in 60 seconds. Using the mean and standard deviation (SD) from the baseline evaluation of all participants, raw scores were converted to *z* scores. For the Concept Shifting Test score, the *z* scores were multiplied by −1, so that positive *z* scores correspond to better performance. The *z* scores of the four neuropsychological tests were then averaged to construct a global cognitive function composite score.

The 23-item Alzheimer’s Disease Cooperative Study – Activities of Daily Living scale, completed by a study partner, measured the ability of the participant to perform baseline and instrumental activities during the prior month [20]. Total scores ranged from 0 (nonperformance or need for extensive help) to 78 (independent performance). The Clinical Dementia Rating – Sum of Boxes gave a global clinical impression of the participant and total scores ranged from 0 (no impairment) to 18 (severe impairment) [21,22].

Safety assessments included the examination of patient medical history, the recording of (serious) adverse events, concomitant medication and nutritional supplement use, and the monitoring of vital signs and safety laboratories for liver function, renal function, and coagulation (at selected sites only). An Independent Data Monitoring Committee and the study medical monitor reviewed adverse events. Serious adverse events were reviewed by the Institutional Review Board of each site. Product intake as recorded in a study partner-supervised patient-reported diary on a daily basis was used to measure product compliance. Study product compliance was calculated as the percentage of product used throughout the study period as compared with the prescribed dosage.

Nutritional blood parameters were docosahexaenoic acid and eicosapentaenoic acid fractions in erythrocyte membranes along with plasma vitamin E and homocysteine levels. Venous blood samples were taken, with a maximum of 30 ml in total per participant for each of the baseline and end-of-study visits, were processed and were stored in a −80°C freezer until batch shipped on dry ice. After extracting lipids from erythrocyte membranes, the fatty acid profile in erythrocyte membranes was assayed by gas chromatography. Plasma vitamin E levels were measured using high-performance liquid chromatography to determine the content of alpha-tocopherol. Homocysteine levels were measured using high-performance liquid chromatography with fluorescence detection after preparing a derivate.

### Sample size

Sample size calculation was based on the repeated measurement design with an estimated difference between the groups of 0 points at baseline, of 0.95 points after 12 weeks of intervention, and of 1.9 points after 24 weeks of intervention with a SD of difference of 10 and an average within-subject correlation of 0.80 over time. Using a type I error of 0.05, a power of 80% and assuming a 15% drop-out rate, this resulted in a sample size of 500 randomized patients. A pre-specified, blinded, re-estimation of the nuisance parameters was conducted for 474 participants to assess whether the calculated sample size was adequate. Based on review of these data along with safety information by the Independent Data Monitoring Committee and the Steering Committee, the study was continued without change using the originally calculated sample size.

### Statistical analysis

Efficacy analyses were performed for the intent-to-treat cohort, including all randomized subjects. Safety analyses were performed for the all-subjects-treated sample (that is, all randomized subjects who received at least one unit of the study product).

Efficacy analyses utilized mixed models for repeated measures. Time was included in the model as a continuous variable using a 24-week period as the unit and with the value 0 at baseline. The model included random intercepts and random slopes for time. The fixed effects of the model consisted of the treatment group, the linear effect of time, and the interaction of treatment group and time. An effect of the treatment group is indicated by statistical significance of the treatment by time interaction. The model takes baseline measurements into account by including them in the outcome vector. Models were then repeated with adjustments for pre-specified confounders (that is, age, gender, education level, type of AD medication, baseline MMSE score, and presence of an apolipoprotein ϵ4 allele). If model assumptions of normality, independence, and constant variance of errors were not adequately met, nonparametric alternatives were used. All statistical analyses were performed using SAS 9.2 (SAS Institute Inc,. Cary, North Carolina, USA). All statistical tests were two-tailed at the 0.05 level of significance.

## Results

### Participant flow

**Figure 1:**
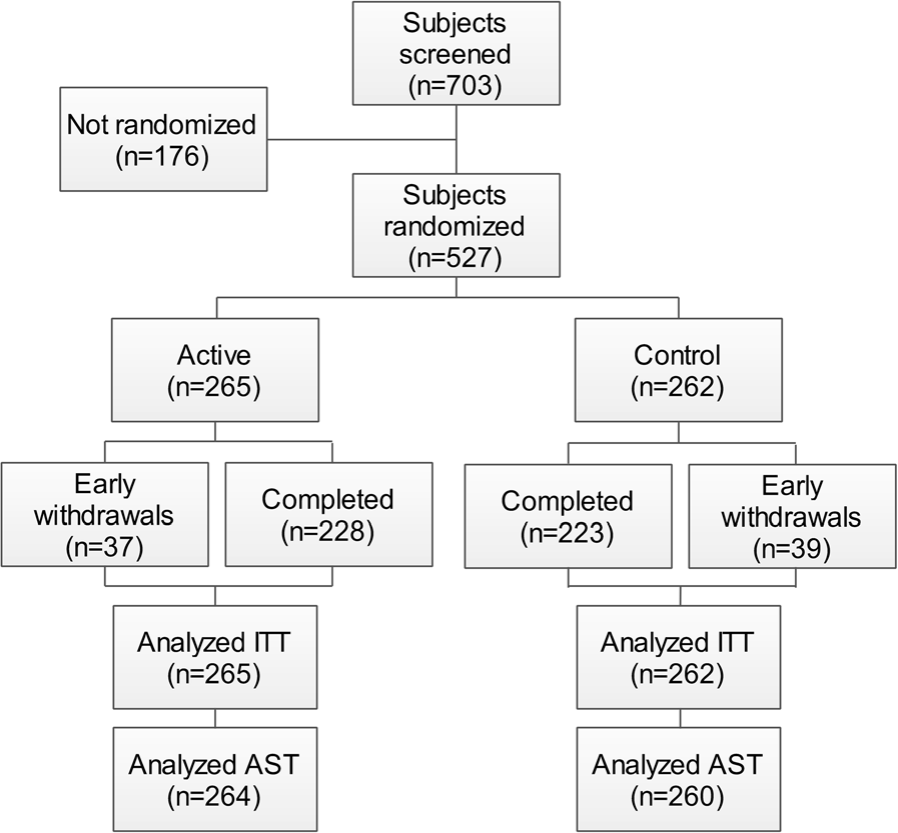
**Flow of participants in the trial.** AST, all subjects treated; ITT, intent to treat.

The trial was conducted between 26 March 2009 and 3 March 2011, including 18 months of recruitment. Of the 703 participants who consented, 167 were excluded because they did not meet the inclusion criteria and nine withdrew from the study prior to randomization (Figure 1). The resulting 527 participants were randomized to Souvenaid (active product, *n* = 265) or control product (*n* = 262). Compared with the intent-to-treat sample, three subjects were excluded from the all-subjects-treated population because they had not taken any study product. Of the 527 subjects who were randomized, 76 (14.4%) withdrew from the study early (*n* = 37 (14.0%) subjects from the active study group; *n* = 39 (14.9%) subjects from the control group).

Baseline characteristics are summarized in Table 1. Randomized participants had a mean age of 76.7 years (SD = 8.2), and a mean education level (defined as number of years after finishing primary school) of 6.5 years (SD = 3.5). Women comprised 52% of the cohort and 94% of participants were White (including Hispanics). The mean time from initial AD diagnosis was 33.8 months (SD = 27.4). The mean duration of AD medication use was 30.1 months (SD = 25.9); 34% of participants were taking an acetylcholinesterase inhibitor agent only, 6% were taking memantine only, and 60% were on both treatments. The mean baseline ADAS-cog score was 23.6 (SD = 9.5) and the mean baseline MMSE was 19.5 (SD = 3.1). Baseline participant characteristics of the cohort did not differ significantly by study group (Table 1).

**Table 1:** Baseline participant characteristics by study group

**Characteristic**	**Active product**	**Control product**
	**(*n*** **= 265)**	**(*n*** **= 262)**
Demographics		
Age (years)	76.6 (8.2)	76.9 (8.2)
Female	139 (52%)	135 (52%)
Education after finishing primary school	6.7 (3.6)	6.4 (3.5)
White (including Hispanic)	250 (94%)	247 (94%)
Mean time from initial AD diagnosis (months)	32.7 (25.0)	34.9 (29.6)
Duration of AD medication use (months)	28.8 (22.9)	31.5 (28.7)
Type of AD medication used		
Acetylcholinesterase inhibitor	87 (32.8%)	92 (35.1%)
Memantine	13 (4.9%)	19 (7.3%)
Acetylcholinesterase inhibitor and memantine combined	164 (61.9%)	151 (57.6%)
Body mass index (kg/m^2^)	26.2 (4.5)	26.6 (4.6)
Mini-Mental State Examination score (out of 30)	19.5 (3.2)	19.4 (3.0)
Presence of apolipoprotein E ϵ4 allele		
No	87 (39.2%)	84 (42.0%)
Yes	135 (60.8%)	116 (58.0%)
Unknown	43	62

Data presented as mean (standard deviation) or number (%). AD, Alzheimer’s disease.

### Primary outcome measure

**Figure 2:**
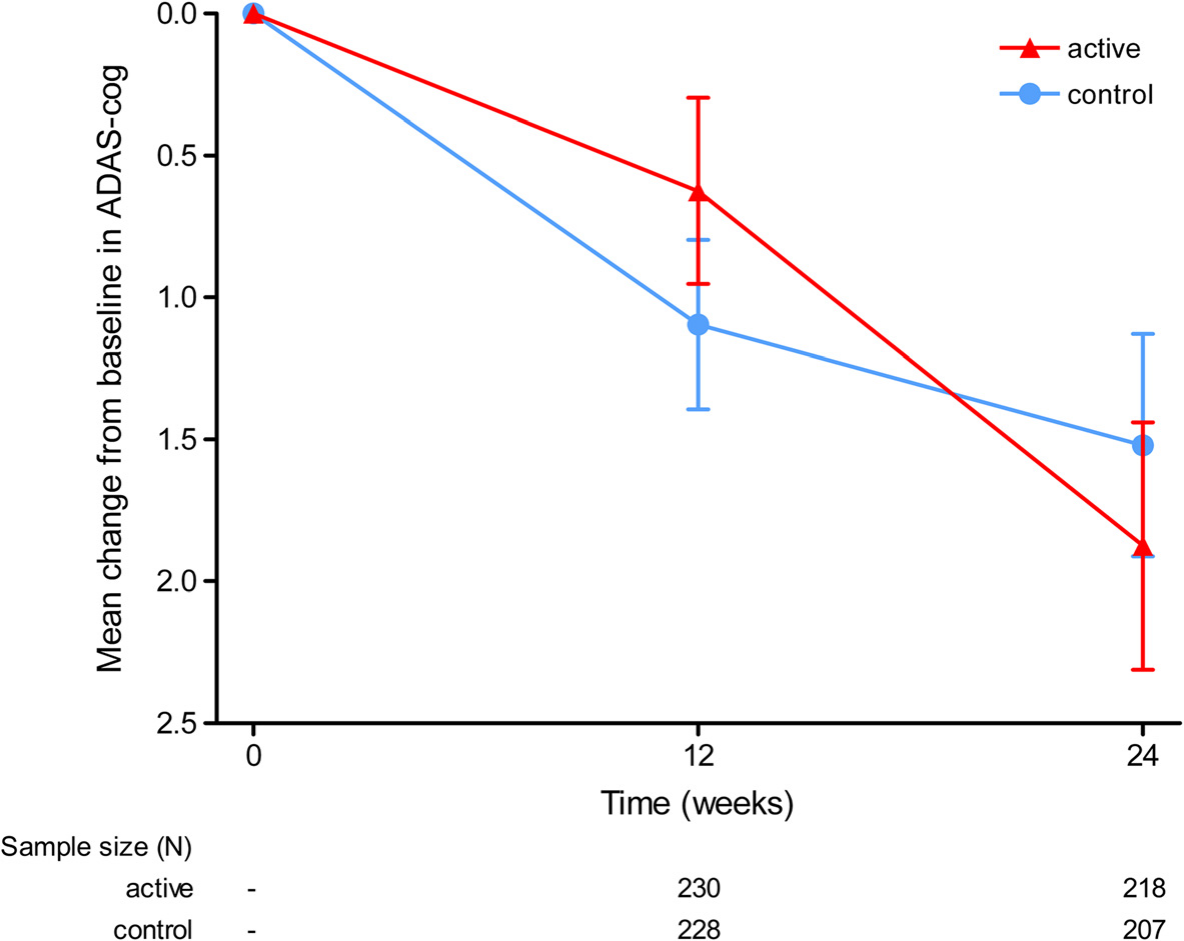
**Mean change from baseline in the Alzheimer’s Disease Assessment Scale – Cognitive Subscale score.** Effects of 24-week intake of study product on the Alzheimer’s Disease Assessment Scale – Cognitive Subscale (ADAS-cog) in the intent-to-treat cohort. Error bars represent standard errors. *P* = 0.513 (mixed models for repeated measures).

ADAS-cog data are presented in Table 2 and Figure 2. ADAS-cog scores showed an increase over time in both study groups, indicating cognitive decline, without significant differences between the active and control group over 24 weeks (between-group difference of 0.37 points, standard error = 0.57, *P* = 0.513, mixed models for repeated measures). The conclusions were unchanged in a subsequent model that corrected for pre-specified confounders.

**Table 2:** Descriptive statistics for ADAS-cog, cognitive test battery, ADCS-ADL and CDR-SB (intent-to-treat cohort)

	**Active product**	**Control product**	***P *** **value**^**a**^
ADAS-cog			
Baseline	23.89 ± 9.59 (258)	23.39 ± 9.34 (257)	0.550
Week 24	25.44 ± 11.56 (220)	24.42 ± 10.95 (208)	0.349
∆ week 24 – baseline	1.88 ± 6.44 (218)	1.52 ± 5.63 (207)	0.547 (0.513)
Cognitive Test Battery, *z* score			
Baseline	0.08 ± 0.75 (228)	−0.02 ± 0.71 (235)	0.153
Week 24	0.09 ± 0.74 (182)	0.01 ± 0.71 (182)	0.260
∆ week 24 – baseline	−0.10 ± 0.47 (179)	−0.05 ± 0.40 (178)	0.301 (0.323)
ADCS-ADL total score			
Baseline	57.95 ± 13.36 (265)	57.38 ± 13.37 (262)	0.623
Week 24	54.66 ± 15.56 (228)	54.15 ± 15.91 (223)	0.731
∆ week 24 – baseline	−3.74 ± 9.76 (228)	−3.66 ± 8.03 (223)	0.926 (0.767)
CDR-SB			
Baseline	6.18 ± 3.01 (264)	6.45 ± 2.89 (259)	0.296
Week 24	6.89 ± 3.35 (227)	7.01 ± 3.41 (223)	0.709
∆ week 24 – baseline	0.77 ± 1.96 (226)	0.69 ± 1.90 (222)	0.676 (0.500)

Data presented as mean ± standard deviation (*n*). ADAS-cog, Alzheimer’s Disease Assessment Scale – Cognitive Subscale, ADCS-ADL, Alzheimer’s Disease Cooperative Study Activity of Daily Living; CDR-SB, Clinical Dementia Rating scale – Sum of Boxes; ∆, difference. ^a^*P* values from *t* tests, active product versus control product, except for values in parentheses that are *P* values from mixed model for repeated measures.

### Secondary outcome measures

**Figure 3:**
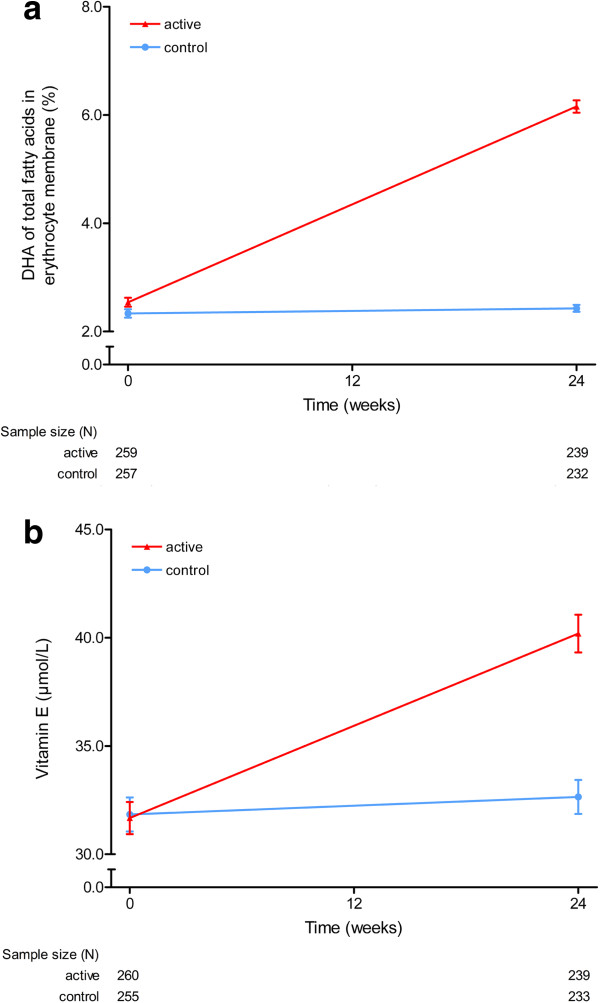
**Mean docosahexaenoic acid and plasma vitamin E levels.** Effects of 24-week intake of study product on **(a)** the percentage docosahexaenoic acid (DHA) of total fatty acids in erythrocyte membrane and **(b)** plasma vitamin E levels (μmol/l) in the intent-to-treat cohort. Error bars represent standard errors. *P* <0.001 (Mann–Whitney U test).

No differences between study groups were observed over 24 weeks in performance on the cognitive test battery, the Alzheimer’s Disease Cooperative Study – Activities of Daily Living, and the Clinical Dementia Rating – Sum of Boxes (Table 2). Mean compliance was 94.1% (SD = 11.9) for the active group and 94.5% (SD = 13.2) for the control group (*P* = 0.689 for between-group difference, *t* test). A significant uptake of docosahexaenoic acid (Figure 3a) and eicosapentaenoic acid into the erythrocyte membranes, increased plasma vitamin E levels (Figure 3b) and decreased homocysteine levels were observed for the active group compared with the control group over the 24-week intervention period (*P* <0.001, Mann–Whitney U test).

### Safety and tolerability

The 24-week study completion rate was 86% (*n* = 228) in the group receiving active product and 85% (*n* = 223) in the control group. In the active group 458 adverse events were reported in 150 participants, compared with the report of 445 adverse events in 165 participants in the control group (*P* = 0.130, Fisher’s exact test, comparing percentage of subjects with at least one adverse event). The number and proportion of patients experiencing one or more adverse events are summarized by body system in Table 3. No significant or relevant differences in adverse events by body system were found. In the active group 34 serious adverse events in 27 subjects were reported, compared with 36 serious adverse events in 34 subjects in the control group. None of the serious adverse events were considered to be related to the use of the study product, except for one serious adverse event (confusion) that was considered to possibly be related to the use of the study product (control). Six serious adverse events resulted in premature discontinuation of the study, including two serious adverse events in the active group (fall and intracranial hemorrhage) and four serious adverse events in the control group (fall, myocardial infarction, nonsmall-cell metastatic lung cancer and malignant mesothelioma), of which malignant mesothelioma resulted in death of the patient. No clinically relevant differences between study groups in vital signs and in blood parameters were noted.

**Table 3:** **Participants experiencing one or more adverse events, by affected body system (all-subjects-treated cohort)**^**a**^

**Body system**	**Active product**	**Control product**	***P *** **value**^**b**^
Total participants	264	260	
Body as a whole	24 (9.1)	33 (12.7)	0.208
Back pain, leg pain, syncope			
Central and peripheral nervous system disorders	27 (10.2)	21 (8.1)	0.450
Headache, dizziness			
Gastrointestinal system disorders	41 (15.5)	38 (14.6)	0.808
Diarrhea, vomiting, nausea			
Metabolic and nutritional disorders	19 (7.2)	19 (7.3)	>0.99
Vitamin D deficiency, hypercholesterolemia, hypokalemia			
Musculoskeletal system disorders	24 (9.1)	15 (5.8)	0.183
Arthralgia, fracture			
Psychiatric disorders	32 (12.1)	43 (16.5)	0.170
Anxiety, agitation, depression, confusion			
Respiratory system disorders	50 (18.9)	42 (16.2)	0.423
Pharyngitis, upper respiratory tract infection			
Skin and appendage disorders	8 (3.0)	18 (6.9)	0.045
Pruritus, increased sweating			
Urinary system disorders	25 (9.5)	19 (7.3)	0.432
Urinary tract infection, urinary incontinence			
Other	20 (7.6)	27 (10.4)	0.287
Fall, surgical intervention			

Data presented as number (%). ^a^For body systems where >5% of participants in either study group reported an event. If a participant experienced the same event more than once within the same body system, the participant was only counted once for the statistical analysis. Adverse events occurring in <5% of patients were: application site disorders, endocrine disorders, hearing and vestibular disorders, heart rate and rhythm disorders, liver and biliary system disorders, myocardial/endocardial/pericardial and valve disorders, neoplasm, platelet, bleeding and clotting disorders, red blood cell disorders, reproductive disorders (male), resistant mechanism disorders, special senses disorders, vascular (extracardiac) disorders, vision disorders, white cell and reticuloendothelial disorders. ^b^Fisher’s exact test.

## Discussion

In this clinical trial of persons with mild-to-moderate AD on stable treatment with available AD medications, the addition of daily oral intake of Souvenaid did not result in 24-week changes in cognitive function, functional abilities, or global clinical impression. Souvenaid was safe and well tolerated and compliance was high, which was confirmed by significantly marked changes in nutritional blood parameters.

Secondary analysis of the proof-of-concept study with Souvenaid [9] pointed to a potential benefit on ADAS-cog in patients with higher ADAS-cog scores (more impaired cognition) at baseline. However, the current, adequately powered clinical trial did not demonstrate an effect on cognition in patients with mild-to-moderate AD receiving AD medication. In the S-Connect study, both the treatment and control groups showed a moderate increase of ADAS-cog scores, suggesting cognitive deterioration, which was consistent with expectations in a population of mild-to-moderate AD patients [23]. Why the active product did not result in slowing cognitive decline in the current study population of persons treated for mild-to-moderate AD is not certain. One potential reason is that a nutrition intervention targeting synaptogenesis may favor earlier use in (very) mild dementia due to AD [8,10] or in pre-dementia stages of AD. In the past decade, clinical trials with nutritional interventions as well as AD drugs and biologics have failed to show benefits in slowing cognitive decline in mild-to-moderate AD. A leading hypothesis for these outcomes has been that the patients in the studies were too far down the pathologic cascade when the neuronal damage and synaptic dysfunction accumulated to an irreversible degree. The hypothesis for the mechanism of action for the current active product is based on its effect on synaptogenesis [24]. Synaptic dysfunction and synapse loss are key hallmarks of AD [25,26], which are present in the very early stage of the disease, even before the emergence of clinical symptoms [27], and strongly correlate with cognitive deterioration [28]. The production of synapses requires neurons, so the potential to benefit from synaptogenesis may be limited in a more moderate stage of dementia due to AD as compared with (very) mild dementia due to AD because of the higher levels of neurodegeneration. In more moderate stages of the clinical syndrome associated with AD, the amount of synaptic dysfunction present may overwhelm potential benefits of Souvenaid on synaptic membrane formation. Current intervention strategies targeting amyloid-beta also are being redirected from mild to moderate AD to asymptomatic or early symptomatic stages of AD [29]. Similarly, the use of Souvenaid may be more beneficial in patients who start nutritional intervention in an earlier stage of the AD disease process, when the neurodegenerative damage is still limited, and thus with greater possibilities to delay cognitive decline. An alternate reason for the study findings is that Souvenaid may not convey a benefit on top of the use of currently available symptomatic pharmacologic therapies in the more moderate stages of the disease. Proving this hypothesis requires further investigation of Souvenaid in drug-naïve patients with moderate AD.

The main strength of the present study is that it provides an informative null regarding add-on therapy with Souvenaid in slowing cognitive decline in a more advanced stage of dementia due to AD. The study was powered adequately to detect a difference between treatment groups on cognitive function. Compliance with active product was high; there were no significant or relevant differences in the adverse event profile and proportion of subjects discontinuing the study due to adverse events between the active product and control groups; the overall drop-out rate (14.4%) was slightly lower than anticipated *a priori*; and the active product resulted in the predicted change in peripheral nutritional blood biomarkers. Also, a rigorous trial design with similar endpoints and safety measures as conducted in pharmaceutical drug trials for regulatory approval was utilized. A limitation of this clinical research study was the inability to determine whether the null result clearly was due to the active product not being effective in the moderate stages of dementia due to AD or was due to not having an additional effect on top of currently approved pharmacological therapies. Also, there was no continuing training program on the cognitive batteries in order to minimize the risk of testing drift during the course of the clinical trial.

This study is part of the Souvenaid clinical trial program that started in 2006 and was based on years of preclinical research examining how specific nutrients may support synaptic function [5]. The multidecade effort to understand the role of nutrients involved in the Kennedy pathway continues to provide insights to help researchers and clinicians better understand the nuanced application of Souvenaid in AD. The null results from the current study in combination with the two other completed clinical trials that showed an effect on memory performance in drug-naïve persons in mild stages of AD [8,10] have led to the focus on use of Souvenaid for cognitive function in the very early stages of the disease. Other randomized controlled trials to obtain more information on the mode of action and long-term efficacy of Souvenaid currently are ongoing, including the 24-month European Union-funded LipiDiDiet study (Dutch Trial Register #NTR1705) in prodromal AD.

## Conclusion

This S-Connect clinical trial establishes the fact that Souvenaid as an add-on intervention does not slow overall cognitive decline and is safe and well tolerated in persons with mild-to-moderate AD using AD medication.

## Abbreviations

AD: Alzheimer’s disease; ADAS-cog: Alzheimer’s Disease Assessment Scale – Cognitive Subscale; MMSE: Mini-Mental State Examination; SD: Standard deviation.

## Competing interests

Study design and planning were carried out in conjunction with the sponsor, Nutricia Research, on behalf of Nutricia Advanced Medical Nutrition. The sponsor also provided the study products and funding for the research, data collection and analysis. The corresponding author had final responsibility for the decision to submit for publication. RCS serves on the Board of Directors of the Alzheimer’s Association – Greater Illinois Chapter; serves as a member of the Investigator Consultation Network for Merck Research Laboratories; served on a research advisory panel for Accera, Inc., and a clinical advisory panel for Nutricia, Inc.; receives or recently received research support as Site Principal Investigator or Site Subinvestigator from Ceregene, Inc., Eisai, Inc., Eli Lilly, Inc., Elan Pharmaceuticals, Inc., Genentech, Inc., Merck & Co., Inc., Metabolic Solutions Development Company, Pamlab, L.L.C., and Pfizer, Inc.; and receives research support from the National Institutes of Health (NIH) (P30 AG101061 (Education and Information Transfer Core Leader), U01 AG010483 (Site Investigator), U01AG024904 (Site Co-investigator), U01 AG029824 (Coinvestigator), and P20MD006886 (Community Outreach/Engagement Core Co-Leader), and from the Illinois Department of Public Health Alzheimer’s Disease Assistance Center. SL reports no financial disclosures relevant to this work. DAB receives research support from the National Institutes of Health, the State of Illinois Excellence in Academic Medicine Act, and Nutricia, Inc.; and has served as a consultant for Nutricia, Inc., Eli Lilly, Inc., and Enzymotic, Ltd. CHS serves on the advisory board and speaker’s bureaus for Novartis International AG, Eli Lilly, Inc., Forest Pharmaceuticals, Inc., and Accera, Inc. JQ receives research support from the NIH(P30 AG008017). SAR serves on the Medical and Scientific Advisory Board of the Alzheimer’s Association – Greater Indiana Chapter and reports no financial disclosures relevant to this work. PS is employed by VU University Medical Center, Amsterdam, which received unrestricted funding from Nutricia Research in the past. PJK, RLW, SHS and AB are employees of Nutricia Research. PS is co-Editor-in-Chief of *Alzheimer’s Research & Therapy* and receives an annual honorarium for the Alzheimer Center at the VU University Medical Center, Amsterdam*.*

## Authors’ contributions

RCS, CHS, SAR, JQ and DAB contributed as investigators to this study. The protocol design and interpretation and statistical analyses of the data were supported by expertise from RCS, PJK, SL, SHS, AB, RLW, DAB and PS. RCS and SL had full access to the entire dataset and performed an independent, blinded analysis of the dataset. All authors have been involved in the drafting or critical revision of the manuscript and approved the final manuscript.

## Supplementary Material

Additional file 1: Table S1 Presenting the nutritional composition of Souvenaid and control product. Click here for file
