# Supplementary material for: The S-Connect study: results from a randomized, controlled trial of Souvenaid in mild-to-moderate Alzheimer’s disease
Source: Alzheimers Res Ther. 2013 Nov 26;5(6):59. doi: 10.1186/alzrt224 (PMC3978853; doi:10.1186/alzrt224)
Supplement: Additional file 1: Table S1 — Presenting the nutritional composition of Souvenaid and control product. [file alzrt224-S1.doc]

**Additional file 1 Table S1. Nutritional composition of 125 mL Souvenaid and 125 mL control product.**

| **Component** | **Souvenaid** | **Control** |
| --- | --- | --- |
| ***Macronutrients*** |  |  |
| Energy, kcal | 125 | 125 |
| Protein, g | 3.8 | 3.8 |
| Carbohydrate, g | 16.5 | 16.5 |
| Fat, g | 4.9 | 4.9 |
| ***Fortasyn Connect*** |  |  |
| EPA, mg | 300 | 0 |
| DHA, mg | 1200 | 0 |
| Phospholipids, mg | 106 | 0 |
| Choline, mg | 400 | 0 |
| UMP (uridine monophosphate), mg | 625 | 0 |
| Vitamin E (alpha-TE), mg | 40 | 0 |
| Vitamin C, mg | 80 | 0 |
| Selenium, mcg | 60 | 0 |
| Vitamin B12, mcg | 3 | 0 |
| Vitamin B6, mg | 1 | 0 |
| Folic acid, mcg | 400 | 0 |
| ***Minerals*** |  |  |
| Sodium, mg | 125 | 125 |
| Potassium, mg | 187.5 | 187.5 |
| Chloride, mg | 156.3 | 156.3 |
| Calcium, mg | 100 | 100 |
| Phosphorus, mg | 87.5 | 87.5 |
| Magnesium, mg | 25.0 | 25.0 |
| ***Other trace elements*** |  |  |
| Iron, mg | 2 | 2 |
| Zinc, mg | 1.5 | 1.5 |
| Iodine, mcg | 16.3 | 16.3 |
| Manganese, mg | 0.41 | 0.41 |
| Copper, mcg | 225 | 225 |
| Molybdenum, mcg | 12.5 | 12.5 |
| Chromium, mcg | 8.4 | 8.4 |
| ***Other vitamins*** |  |  |
| Vitamin A, mcg | 200 | 200 |
| Thiamin (B1), mg | 0.19 | 0.19 |
| Riboflavin (B2) , mg | 0.20 | 0.20 |
| Niacin (B3), mg NE | 2.25 | 2.25 |
| Pantothenic acid (B5), mg | 0.66 | 0.66 |
| Vitamin D, mcg | 0.88 | 0.88 |
| Biotin, mcg | 5.0 | 5.0 |
| Vitamin K, mcg | 6.6 | 6.6 |

*Note.* Abbreviations: EPA, eicosapentaenoic acid; DHA, docosahexaenoic acid; TE, tocopherol equivalents; NE, niacin equivalents.
